# A Stepwise Anatomy-Based Protocol for Total Laparoscopic Hysterectomy: Educational Tool with Broad Clinical Utility

**DOI:** 10.3390/diagnostics15141736

**Published:** 2025-07-08

**Authors:** Rudolf Lampé, Nóra Margitai, Péter Török, Luca Lukács, Mónika Orosz

**Affiliations:** Department of Obstetrics and Gynecology, Faculty of Medicine, University of Debrecen, 98 Nagyerdei krt., 4032 Debrecen, Hungary; margitai.nora@med.unideb.hu (N.M.); torok.peter@med.unideb.hu (P.T.); lukacs.luca@med.unideb.hu (L.L.); orosz.monika@med.unideb.hu (M.O.)

**Keywords:** total laparoscopic hysterectomy, surgical standardization, uterine artery ligation, retroperitoneal dissection

## Abstract

**Background:** Total laparoscopic hysterectomy (TLH) is widely accepted as the preferred minimally invasive technique for the treatment of benign gynecologic conditions. However, significant heterogeneity persists in the literature regarding the operative sequence, particularly for steps such as uterine artery ligation, ureteral identification, and vaginal cuff closure. This lack of standardization may affect complication rates, reproducibility in surgical training, and procedural efficiency. The objective of this study was to develop and evaluate a standardized, anatomically justified surgical protocol for TLH primarily designed for training purposes but applicable to most clinical cases. **Methods:** This retrospective observational study analyzed 109 patients who underwent TLH between January 2016 and July 2020 at a single tertiary care center. A fixed sequence of surgical steps was applied in all cases, emphasizing early uterine artery ligation at its origin, broad ligament fenestration above the ureter, and laparoscopic figure-of-eight vaginal cuff closure. Patient demographics, operative data, and perioperative outcomes were extracted and analyzed. **Results:** The mean operative time was 67.2 ± 18.4 min, and the mean uterine weight was 211.9 ± 95.3 g. Intraoperative complications were observed in 3.7% of cases and included bladder injury in 1.8% and small bowel injury in 1.8%, all of which were managed laparoscopically without conversion. Vaginal cuff dehiscence occurred in 1.8%, and postoperative vaginal bleeding in 3.7% of patients. One patient (0.9%) required reoperation due to a vaginal cuff hematoma/abscess. No postoperative infections requiring intervention were reported. The mean hemoglobin drop on the first postoperative day was 1.2 ± 0.9 g/dL. **Conclusions:** Our findings support the feasibility, reproducibility, and safety of a structured TLH protocol based on anatomical landmarks and early vascular control. Widespread adoption of similar protocols may improve consistency and training, with broad applicability in routine surgical practice and potential adaptation in severely complex cases; however, further validation in multicenter studies is warranted.

## 1. Introduction

Total laparoscopic hysterectomy (TLH) has become a widely accepted surgical technique for the treatment of benign and malignant gynecological conditions. Its minimally invasive nature provides numerous benefits, including reduced postoperative pain, shorter hospital stay, and faster recovery when compared to abdominal approaches [1,2]. However, despite the widespread adoption of TLH, there remains a significant lack of consensus regarding the standardization of its operative steps, which can result in variability in surgical outcomes, training inconsistencies, and challenges in quality control [3,4].

Standardization of surgical procedures has been shown to improve patient safety, facilitate teaching, and reduce operative time and complications [5]. Yet, in TLH, the definition and execution of critical steps—such as bladder dissection, uterine artery ligation, or colpotomy—differ significantly across institutions and publications. For instance, while in [3] authors describe a 10-step method emphasizing early identification of the uterine artery, other authors propose variations in the sequence or techniques used for vascular sealing and dissection [5]. Similarly, vaginal cuff closure techniques vary not only in method—laparoscopic vs. transvaginal—but also in suturing style, which may impact postoperative healing and complications [6,7].

Such discrepancies underscore the necessity for a literature-supported and data-informed standard surgical protocol. A widely applicable, anatomically structured operative pathway could enhance surgical training, improve inter-institutional comparability, and support better patient outcomes, while still allowing flexibility for case-specific adaptations. To address these discrepancies, we developed and implemented a stepwise, anatomy-based surgical protocol for TLH, which we now present with its anatomical rationale and a retrospective evaluation based on our institutional experience. The protocol is intended primarily as an educational framework. While not designed for universal application, the protocol has proven broadly applicable across routine cases in our experience.

While this protocol was originally developed as a teaching model, we found it to be applicable in most routine TLH procedures. Only in cases of severe complexity—such as those involving extensive adhesions or advanced endometriosis—may certain steps require modification.

## 2. Patients and Methods

This retrospective observational study was conducted at the Department of Obstetrics and Gynecology, University of Debrecen, between January 2016 and July 2020.

Patients were included if the entire surgical procedure followed the standardized steps described below, pre- and postoperative blood tests were available, and the vaginal cuff was closed laparoscopically. Postoperative complications were recorded up to the 6-week follow-up visit.

Clinical data were collected from our institutional electronic surgical database, which logs all operative gynecologic procedures. The surgical protocol was developed prospectively as part of an institutional effort to standardize TLH training and promote anatomical consistency among surgeons. However, the present study was designed and conducted retrospectively to evaluate outcomes of procedures performed under this protocol. The initial cohort of 216 TLH cases was identified retrospectively. Exclusion criteria included malignant pathology, transvaginal cuff closure, severe intraoperative adhesions, deep infiltrating endometriosis, deviation from the protocol, and missing follow-up data. After applying exclusion criteria, a total of 109 cases were included in the final analysis. Collected patient characteristics included age, body mass index (BMI), parity, number of cesarean and vaginal births, comorbidities (e.g., hypertension, diabetes mellitus, chronic obstructive pulmonary disease), surgical indication, and number of previous laparotomies.

Estimated blood loss was approximated by the drop in hemoglobin from preoperative to postoperative day 1, as intraoperative blood loss was not directly measured. Operative time was skin-to-suture time. Vaginal bleeding was defined as any postoperative bleeding that required clinical evaluation beyond routine follow-up, including persistent spotting beyond 10 days, moderate bleeding requiring medical consultation, or presentation to the emergency department. Mild spotting that resolved spontaneously and did not require evaluation was not included.

All 109 procedures were performed by a consistent group of 4–5 experienced gynecologic laparoscopists who had previously trained together in the application of this standardized protocol. While trainees were occasionally present in the operating room as assistants or observers, none of the surgical steps were performed by them.

Postoperative complications were identified through systematic review of follow-up documentation in the electronic medical record, including surgical reports, inpatient notes, discharge summaries, and outpatient follow-up visits up to 6 weeks postoperatively. Complications were classified as major or minor. Major complications were defined as events requiring surgical re-intervention, blood transfusion, or prolonged hospitalization (>4 days). Minor complications included events managed conservatively, such as urinary tract infection or small hematomas. In ambiguous cases, classification was confirmed by consensus of the senior surgical team.

Statistical analysis was primarily descriptive. Categorical variables are reported as frequencies and percentages, while continuous variables are expressed as means with standard deviations (SD) or medians with interquartile ranges (IQR), depending on distribution. No comparative or inferential statistical tests were performed, as the objective was to evaluate outcomes from a single cohort without a control group. Statistical analysis was conducted using SPSS software, version 26.0 (IBM Corp., Armonk, NY, USA).

### 2.1. Preoperative Protocol

All patients underwent preoperative anesthetic evaluation. All patients were instructed to fast for at least 6 h before surgery in accordance with standard anesthesia guidelines. Bowel preparation was not administered routinely, just selectively in cases with elevated risk of intestinal injury. A single dose of 2 g intravenous cefazolin was used in all cases, unless contraindicated by allergy. All patients received thromboprophylaxis with low molecular weight heparin (enoxaparin 40 mg subcutaneously once daily) beginning on the evening of surgery and continued postoperatively. In addition, mechanical prophylaxis (compression stockings and/or intermittent pneumatic compression) was applied intraoperatively and during the early postoperative period.

### 2.2. Operative Setup

Surgeries were performed under general endotracheal anesthesia. Patients were placed in lithotomy position with a 12–15° Trendelenburg tilt. A urinary catheter was inserted before surgery and removed 12 h postoperatively. The operating table was lowered maximally to ensure ergonomic access.

A reusable metal uterine manipulator consisting of a rigid intrauterine rod and a detachable vaginal fornix cup was used in all cases. This economical, sterilizable, and simple device provided stable uterine positioning and reliable anatomical exposure to facilitate safe colpotomy. A 10 mm optical trocar with a 30° angled laparoscope was placed at the umbilicus, by direct entry, followed by the placement of three 5 mm trocars under visual control: two in the lower quadrants (3 cm above the symphysis and 2 cm medial to the anterior superior iliac spine). All procedures were initiated using direct trocar entry at the umbilicus. The Veress needle was not used, as the patients had no contraindications for direct access and the surgeon preferred this technique for speed and reliability. No patients required left upper quadrant entry, as no significant adhesions or prior contraindications were identified during preoperative evaluation. Intra-abdominal pressure was maintained at 15 mmHg.

During all procedures, the surgeon used an ultrasonic cutting device and a bipolar coagulation instrument, while the assistant used blunt grasping forceps. Colpotomy was performed using monopolar energy in pure cutting mode at 40 W with an L-hook electrode, applied circumferentially around the colpotomy cup. Ultrasonic energy was not employed for this step. We opted for monopolar cutting because it provides a clean incision with minimal lateral thermal spread, which facilitates precise entry into the vaginal fornix and optimal healing at the cuff margins. Vaginal cuff closure was performed using two needle holders for intracorporeal suturing using a continuous figure-of-eight technique. Barbed sutures were not used; all closures were performed with standard 0 absorbable braided sutures.

The accompanying video (see Supplementary Materials) provides a detailed depiction of the surgical technique for TLH.

### 2.3. Surgical Technique—Standardized Stepwise TLH Protocol

**Figure 1:**
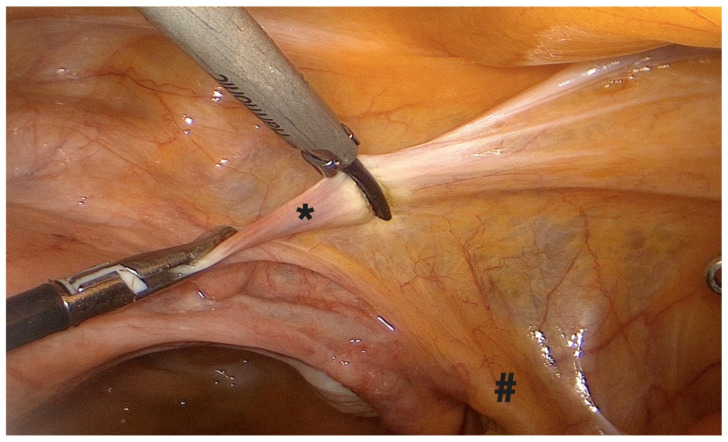
Transection of the round ligament. *: right round ligament, #: right IP ligament.

**Figure 2:**
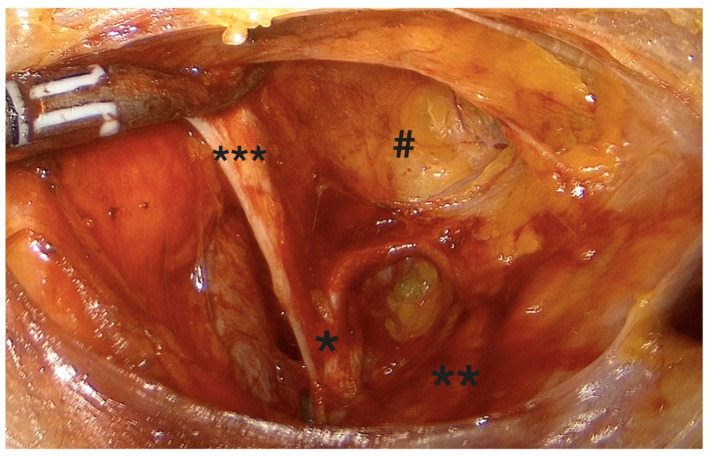
Origin of the uterine artery on the left side. *: uterine artery, **: ureter, ***: hypogastric artery, #: paravesical space.

**Figure 3:**
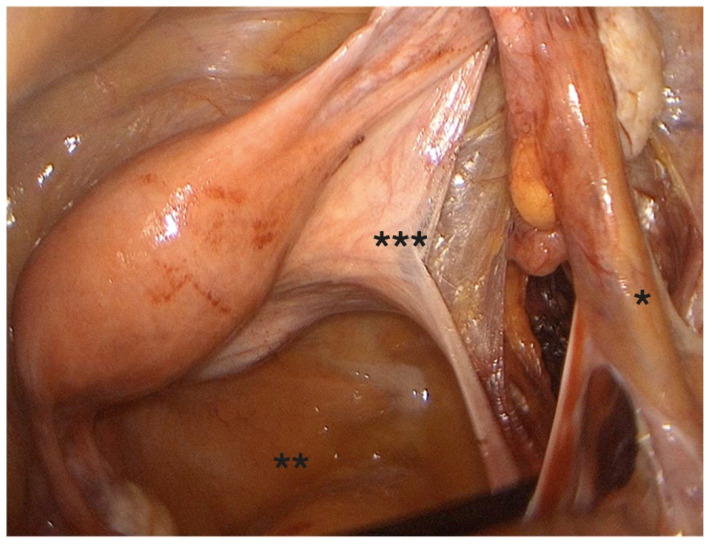
Fenestration of the broad ligament. *: IP ligament, **: pouch of Douglas, ***: broad ligament.

**Figure 4:**
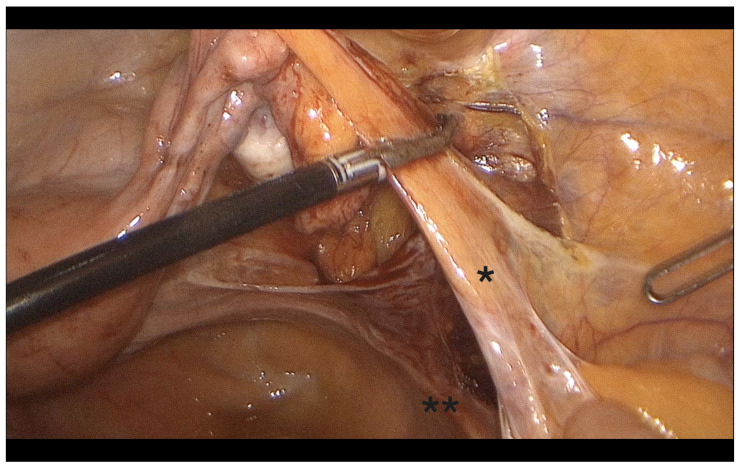
Coagulation of the infundibulopelvic ligament. *: IP ligament, **: ureter behind the peritoneum.

**Figure 5:**
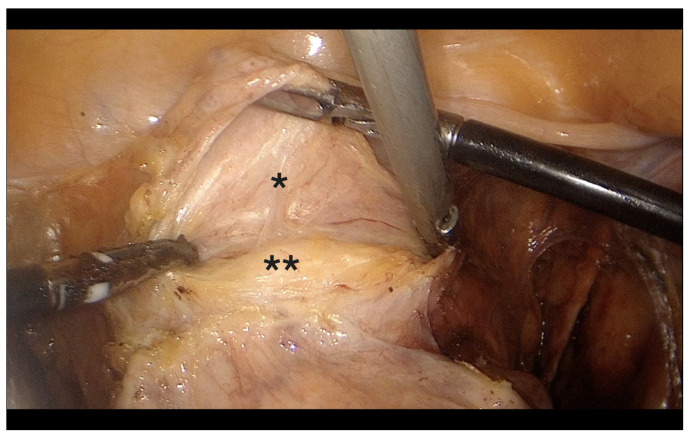
Mobilization of the urinary bladder. *: urinary bladder, **: anterior vaginal wall, with the edge of the uterine manipulator visible behind it, positioned within the anterior vaginal fornix.

**Figure 6:**
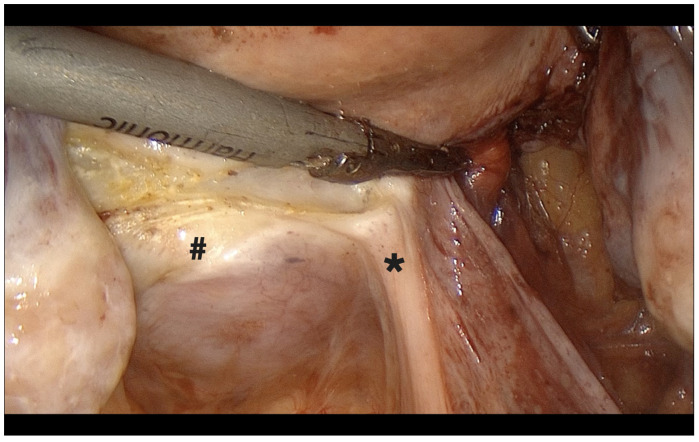
Uterosacral ligament dissection. *: right uterosacral ligament, #: The posterior dissection line, which is defined by the edge of the manipulator, which elevates the posterior vaginal fornix.

**Figure 7:**
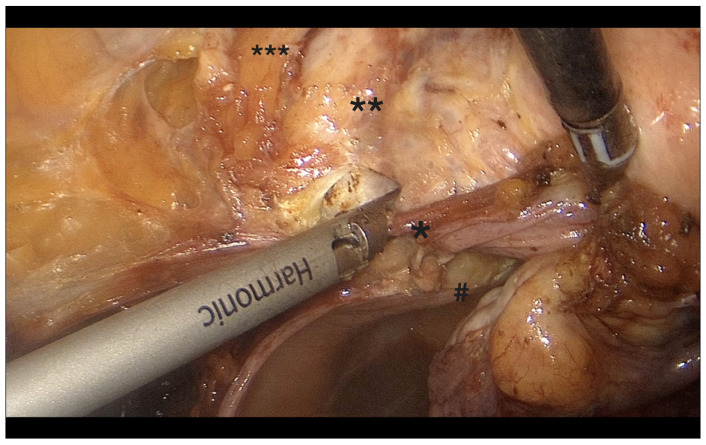
Dissection of the left cardinal ligament, including the distal uterine artery and surrounding connective tissue. *: cardinal ligament, **: anterior vaginal fornix, ***: urinary bladder, #: sacrouterine ligament.

**Figure 8:**
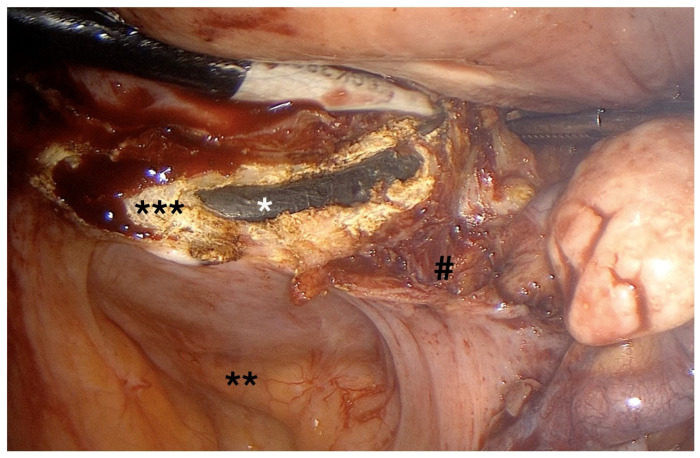
Posterior colpotomy. *: edge of the uterine manipulator, **: pouch of Douglas, ***: posterior vaginal fornix, #: transected uterine vascular structures.

**Figure 9:**
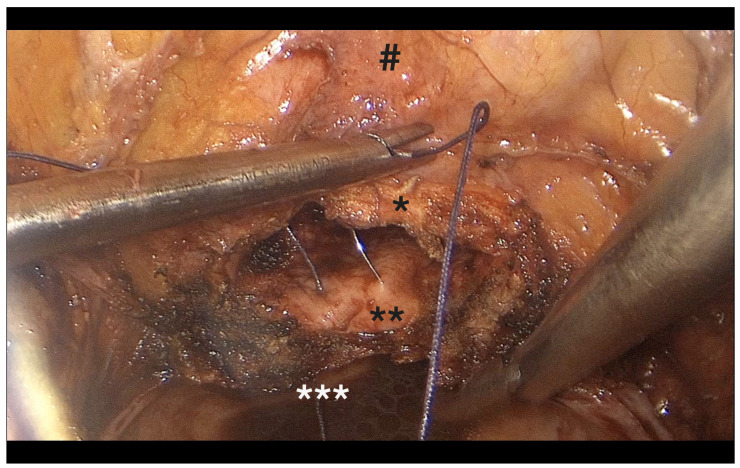
Vaginal closure. *: anterior vaginal wall, **: posterior vaginal wall, ***: left sacrouterine ligament, #: urinary bladder.

**1.**  **Right pelvic sidewall procedures**
  - Coagulation and transection of the round ligament laterally, away from adnexal and iliac vessels (Figure 1).
  - Retroperitoneal dissection was performed for identification of the obliterated umbilical artery and ligation of the uterine artery at its origin from the internal iliac artery, under direct visualization and lateral displacement of the ureter (Figure 2). The uterine artery was first coagulated using bipolar energy and then transected with ultrasonic shears to ensure complete vessel obliteration and hemostasis.
  - Fenestration of the broad ligament above the ureter (Figure 3).
  - Transection of the mesosalpinx and ovarian ligament (or infundibulopelvic ligament in case of adnexectomy, Figure 4).
**2.**  **Left pelvic sidewall procedures**
  - Same steps as on the right side.
**3.**  **Bladder mobilization**
  - Opening of the anterior broad ligament fold; vesicouterine space opened with careful blunt dissection, mobilizing the bladder ~2–3 cm caudally (Figure 5). In patients with prior cesarean section or anterior adhesions, bilateral paravesical space development facilitated safe identification of the vesicouterine plane and minimized bladder injury risk.
**4.**  **Posterior peritoneum and uterosacral ligament dissection**
  - After rotating the optic, uterosacral ligaments were transected under guidance from the uterine manipulator (Figure 6).
**5.**  **Cardinal ligament and distal uterine artery transection**
  - Performed with perpendicular dissection under direct visualization of the manipulator, beginning on the right side (Figure 7).
**6.**  **Colpotomy**
  - Initiated medially on the posterior vaginal wall and extended circumferentially around the cervix, with optic adjustments for visualization (Figure 8).
**7.**  **Uterus extraction**
  - Removed vaginally or by intra-abdominal or vaginal morcellation depending on uterine size.
**8.**  **Vaginal cuff closure**
  - Performed laparoscopically, incorporating mucosa, vesicovaginal fascia, and both uterosacral ligaments (Figure 9).

For ease of reference and didactic clarity, Table 1 presents a structured summary of the proposed TLH protocol. Each step is listed with a concise description and its critical anatomical or technical considerations.

### 2.4. Postoperative Management

After completion of colpotomy and cuff closure, surrounding structures were re-evaluated. Lavage and aspiration were performed, and trocars were removed under direct vision. Skin incisions were closed with absorbable 4-0 monofilament sutures. Routine drain placement was avoided. Postoperative care included analgesics, antiemetics as needed, and continuation of thromboprophylaxis. On the first postoperative day, follow-up blood tests were obtained to evaluate hemoglobin levels. Patients were typically discharged on the second postoperative day if their condition allowed, with follow-up gynecological evaluation scheduled at six weeks.

### 2.5. Outcomes and Data Analysis

The following surgical variables were analyzed: operative time (from skin incision to final suture), estimated blood loss (based on hemoglobin drop from pre-op to postoperative day 1), uterine weight (measured by pathologist), and intra- and postoperative complications. Descriptive statistics were calculated. Continuous variables were expressed as mean ± standard deviation, and categorical variables as frequencies

The study was approved by the Institutional Ethics Committee of the University of Debrecen, approval number: DE RKEB/IKEB H.0202-2020, and conducted in accordance with the Declaration of Helsinki. Written informed consent was waived due to the retrospective nature of the study and anonymization of patient data.

## 3. Results

### 3.1. Patient Characteristics

A total of 216 TLH procedures were performed during the study period. Of these, 107 were excluded based on predefined criteria, leaving 109 cases for final analysis. We excluded cases with malignant pathology (*n* = 43), transvaginal cuff closure (*n* = 8), severe intraoperative adhesions (*n* = 8), deep infiltrating endometriosis (*n* = 7), deviations from the standardized protocol (*n* = 17), and missing follow-up data (*n* = 24). Patient demographic details are provided in Table 2. The mean age of the cohort was 51.1 ± 10.3 years, and the mean body mass index (BMI) was 26.8 ± 5.3 kg/m^2^. Nineteen patients (17.4%) had a BMI greater than 30. The majority of patients (90.8%) had a parity between 1 and 4. Thirty-six patients (33%) were postmenopausal at the time of surgery, and 88 (80.7%) had a history of vaginal delivery. Previous surgical history included laparoscopic procedures in 19 patients (17.4%) and open abdominal surgeries in 38 patients (34.9%).

### 3.2. Surgical Indications and Intraoperative Details

The distribution of surgical indications is summarized in Table 3. The most common indications were abnormal uterine bleeding (39.5% of cases) and uterine fibroids (32.1%), with the remainder including adenomyosis, endometriosis, and other benign conditions. Cases with known or suspected endometriosis were not excluded; however, only patients with stage I–II endometriosis were included. No cases of deep infiltrating or extensive stage III–IV endometriosis were present in the analyzed cohort. The mean uterine weight was 211.9 ± 95.3 g. The average total operative time was 67.2 ± 18.4 min. Adhesiolysis was necessary in 19 cases (17.4%). Although not statistically analyzed due to sample size, we observed qualitatively that cases requiring adhesiolysis tended to have longer operative times, as expected. The mean hemoglobin decrease on postoperative day 1 was 1.2 ± 0.9 g/dL, and the average hospital stay was 2.3 ± 1.1 days. Ten patients (9.2%) required hospitalization beyond 4 days due to postoperative complications or delayed recovery.

### 3.3. Intraoperative and Postoperative Complications

Intra- and postoperative complications are listed in Table 4. Intraoperative complications occurred in 4 patients (3.7%), including 2 bladder injuries (1.8%) and 2 small bowel injuries (1.8%). All intraoperative events were recognized immediately and successfully managed laparoscopically, without the need for conversion to laparotomy. Postoperative complications were observed in a limited number of cases. Vaginal cuff dehiscence occurred in 2 patients (1.8%), and in all cases, the defect was closed transvaginally using absorbable sutures. One patient (0.9%) developed a vaginal cuff hematoma/abscess that required surgical re-intervention (reoperation for drainage). Postoperative vaginal bleeding was documented in 4 patients (3.7%), all of whom recovered spontaneously without requiring additional surgical or pharmacological treatment. Paralytic ileus occurred in 2 patients (1.8%), and both were managed conservatively with bowel rest and supportive care. There were no cases of vaginal evisceration, no postoperative infections requiring treatment, and no conversions to open surgery. One patient (0.9%) developed a postoperative pelvic fluid collection that required laparoscopic drainage due to persistent pain and concern for possible abscess; however, no purulent material was found, and cultures remained negative. This case was classified as a sterile seroma rather than a true infectious complication. Accordingly, it was recorded as a surgical intervention without postoperative infection. Two patients (1.8%) received postoperative blood transfusions due to symptomatic anemia; both had documented preoperative hemoglobin levels below normal thresholds. No ureteral injuries occurred.

## 4. Discussion

This manuscript primarily aims to offer a structured, anatomy-based surgical protocol that serves as a training model. At the same time, the protocol has shown broad applicability in clinical practice and is adaptable to varying levels of surgical complexity.

### 4.1. General Significance of Surgical Standardization in TLH

TLH has become the preferred approach, and it appears to be superior to abdominal hysterectomy for benign gynecologic conditions in many settings due to its benefits in recovery time, pain control, and cosmetic outcome [3,4]. Yet, there is notable heterogeneity in the literature regarding the exact operative steps, with no universally adopted standard.

The absence of clear consensus and strong evidence often leads to variability in clinical decision-making. In such cases, healthcare professionals tend to rely more heavily on individual judgment or institutional traditions. However, there is growing evidence that aligning surgical procedures with standardized, evidence-based protocols improves clinical outcomes and reduces healthcare expenditures. This underlines the current need to establish a well-defined standard of care for laparoscopic hysterectomy, rooted in the principles of evidence-based medicine [4].

### 4.2. Anatomical and Technical Rationale for Each Surgical Step

The standardized steps proposed in our surgical protocol are based on anatomic logic and are supported by evidence from contemporary literature.

The sequence begins with medial coagulation and transection of the round ligament. This initial maneuver is widely recognized as a safe starting point for laparoscopic hysterectomy, facilitating entry into the retroperitoneal space while minimizing the risk of injury to the adnexal vessels.

The pelvic peritoneum is incised parallel to the infundibulopelvic (IP) ligament to access the retroperitoneal space. Dissection proceeds into the medial and lateral paravesical spaces, where the obliterated branch of the internal iliac artery, known as the hypogastric artery (or more precisely, the obliterated umbilical artery), is identified. Traction applied caudally to this structure causes tension on the lateral umbilical ligament along the anterior abdominal wall, thereby confirming its correct identification. By tracing the hypogastric artery proximally, the origin of the uterine artery from the internal iliac artery can be located. The ureter is typically found just medial to this point, crossing beneath the uterine artery. After identifying the ureter within the pararectal space, it is carefully displaced medially to clear the operative field. The uterine artery is then safely coagulated and transected at its origin. A key distinguishing feature of our technique is the ligation of the uterine artery at its origin from the internal iliac artery. Multiple studies have underlined the advantages of this approach, citing reduced intraoperative bleeding and enhanced control over pelvic hemostasis. Shorter operative time and hospital stay were also observed with this technique [8,9,10]. Recent findings also support the hemostatic benefits of laparoscopic arterial control. For example, Moratalla-Bartolomé et al. demonstrated the surgical impact of transient bilateral occlusion of uterine and utero-ovarian arteries during laparoscopic myomectomy [11]. Although their technique differed from our permanent ligation approach, the underlying principle of targeted vascular control is shared.

Following vascular isolation, we perform a broad ligament fenestration above the ureter. This maneuver is critical for improving visualization and ensuring ureteral protection [12].

When proceeding to adnexal structures, the protocol differentiates between ovarian preservation and salpingo-oophorectomy. In ovarian preservation cases, transection of the mesosalpinx and proper ovarian ligament is performed, while salpingo-oophorectomy cases involve ligation of the IP ligament. Because the broad ligament has been fenestrated in a prior step, the risk of cutting too deeply—and consequently the risk of ureteral injury—is minimal. Particular attention was given to dorsally deflecting the ureter before coagulating and transecting the infundibulopelvic ligament to further decrease the risk of injury. The IP ligament should be coagulated meticulously, as it retracts to the retroperitoneal space.

The anterior peritoneal fold is opened to initiate bladder mobilization. Performing this maneuver under optic guidance ensures consistent depth and angle of dissection, particularly in patients with previous cesarean deliveries. The bladder should be pulled upward to be able to separate it from the anterior vaginal wall and to dissect the bladder pillars.

Subsequently, we dissect the posterior peritoneum and uterosacral ligaments after turning the laparoscope 30 degrees upward, a step that provides a clearer angle of approach to deep pelvic structures. The peritoneum is cut from the left to right side. At this point, the surgeon switches instrument hands (holding the ultrasonic scalpel in the nondominant hand and the bipolar forceps in the dominant hand) to facilitate the next steps.

Transection of the cardinal ligaments, including the distal uterine artery and surrounding connective tissue, follows, starting with the left side, followed by the right side, to avoid unnecessary instrument exchange between hands. This step is crucial for preserving a safe trajectory around the uterine vasculature, which becomes clearly visible after incising both the anterior and posterior peritoneum. The uterine manipulator provides effective traction and counter-traction, which is essential for clear visualization throughout these steps.

Colpotomy is initiated from the posterior aspect of the cervix and carried out in a quadrant-based sequence. This strategy improves visualization and allows for stepwise expansion of the colpotomy, particularly in patients with large uteri or distorted anatomy. The posterior colpotomy allows circumferential incision of the vaginal fornix along the edge of the manipulator, even if the manipulator loses its grip on the uterus.

Finally, the vaginal cuff is closed laparoscopically with a conventional 0 absorbable braided suture incorporating the vesicovaginal fascia, vaginal mucosa, and uterosacral ligament on both sides. The purpose of incorporating the uterosacral ligaments during vaginal cuff closure is to provide pelvic support. While this is not supported by high-level evidence, it is commonly practiced and was applied in all cases. There is no established evidence that the use of any particular suture material reduces the rate of postoperative complications. Randomized trials comparing barbed and conventional sutures have found no significant difference in adverse event rates [13]. This closure technique has been associated with a lower rate of dehiscence and better long-term pelvic support.

In sum, the surgical steps we employ are not arbitrary but rather grounded in both anatomical principles and clinical outcomes. Each maneuver builds upon the previous one to create a cohesive, safe, and replicable sequence, which we believe can serve as a reference model for future standardization efforts in TLH.

While our surgical protocol aligns with many contemporary international practices, several key differences distinguish it from the standardized steps proposed by the ESGE (European Society for Gynaecological Endoscopy) 2019 consensus guidelines [14]. These differences reflect both our center’s emphasis on anatomical clarity and the prioritization of vascular and ureteral safety.

Most notably, the ESGE guidelines recommend ligation of the uterine artery at the ascending branch near the uterus, often after posterior peritoneal opening and broad ligament fenestration. In contrast, our approach emphasizes early retroperitoneal dissection and coagulation of the uterine artery at its origin from the internal iliac artery. This modification may require greater technical skill and slightly lengthen the procedure at the beginning, but it enables early ureter identification, reduces intraoperative bleeding, and improves control over deep vascular pedicles, especially in patients with large uteri or distorted pelvic anatomy.

Similarly, in the treatment of adnexal structures, ESGE outlines two optional approaches: one closer to the uterine cornu, and one near the pelvic sidewall. Our method consistently chooses the latter, opening a wide peritoneal window above the ureter, which we believe provides a safer surgical field and better lateral mobilization. The ESGE document acknowledges that fenestration is not always necessary, but we contend that standardizing it minimizes variability in ureteral exposure.

In terms of vaginal cuff closure, both ESGE and our protocol endorse laparoscopic suturing techniques. However, while ESGE lists vaginal closure as an option, our technique mandates a laparoscopic figure-of-eight approach that incorporates both mucosal and fascial layers as well as the uterosacral ligaments.

In summary, while the ESGE working group offers a widely accepted framework in some steps, we believe that our surgical steps introduce refinements that prioritize anatomical safety, reproducibility, and hemostatic precision. Incorporating these elements into routine laparoscopic hysterectomy practice may enhance patient outcomes and surgeon confidence, particularly in more complex pelvic anatomies. While we advocate for a structured, reproducible technique, we fully acknowledge that individual surgical decisions must be guided by intraoperative findings, patient-specific anatomy, and surgeon expertise.

### 4.3. Comparison of Our Clinical Outcomes with International Studies

In our cohort of 109 patients, the mean operative time was 67.2 ± 18.4 min, which is markedly shorter than the median operative times typically reported in large multicenter analyses of TLH [15]. For instance, in national datasets, TLH operative times often range between 89 and 158 min, depending on institutional experience, patient selection, and case complexity. Notably, these higher durations may reflect contributions from diverse surgical teams and robotic-assisted procedures. In contrast, our standardized anatomical approach, performed consistently by experienced surgeons, may have contributed to improved surgical efficiency. This was achieved without compromising procedural safety or completeness, as reflected by low complication rates and satisfactory outcomes across a range of uterine sizes.

Intraoperative complication rates were low (3.7%), with two bladder and one small bowel injuries, and all managed laparoscopically without conversion to laparotomy. These values fall within internationally reported ranges of 4–14% [16,17], similar to the rate of bladder injury (2.9%) reported in a large prospective study [18].

Vaginal cuff dehiscence occurred in 1.8% of cases in our series, slightly above the 1.2–3.4% rates described by others [19,20]. However, all cases were successfully repaired transvaginally, and no episodes of evisceration or major infection were observed.

The mean hemoglobin drop on postoperative day one was 1.2 g/dL, and the transfusion rate was 1.8%. Both transfused patients had preoperative hemoglobin levels below 120 g/L but above 100 g/L, indicating mild anemia. While early uterine artery ligation at its origin does not eliminate intraoperative blood loss, it may help reduce the risk of significant hemorrhage during the initial steps of dissection. The hemoglobin drop on the first postoperative day was 1.2 g/dL, a value slightly lower than those observed in comparable studies, suggesting effective intraoperative hemostasis. The average hemoglobin decrease observed on the first postoperative day was modest and slightly lower than those reported by other authors (1.4–2.1 g/dL) [9,21,22].

Although length of stay was recorded, it does not reflect clinical necessity or Enhanced Recovery After Surgery (ERAS) standards. The average hospital stay of 2.3 ± 1.1 days was primarily attributable to institutional discharge protocols mandating a longer inpatient period, regardless of postoperative recovery status. A total of 10 patients (9.2%) required hospitalization beyond four days, primarily due to paralytic ileus or the need for management of intra- or postoperative complications.

This study has several limitations that should be acknowledged. First, its retrospective design limits the ability to control for potential confounding variables, such as surgeon experience or patient comorbidities. Patients who did not undergo the standardized sequence or had transvaginal cuff closure were excluded from analysis, which may introduce selection bias and limit the generalizability of our findings. All procedures were performed by an experienced surgical team at a single tertiary center. Although the protocol was designed as a training framework, all critical steps were performed by experienced surgeons. Trainees were present but did not perform any key elements of the procedure independently. While the protocol was consistently applied, outcomes may vary across different settings and skill levels. Furthermore, although intraoperative and early postoperative outcomes were comprehensively recorded, long-term follow-up—particularly regarding vaginal cuff integrity, pelvic floor function, or recurrence of symptoms—was not assessed. Finally, while the standardized technique was applied uniformly, we did not perform subgroup analysis based on uterine size, parity, or menopausal status, which may influence surgical complexity and outcomes.

## 5. Conclusions

This study proposes a highly structured and real-world supported TLH protocol, designed to maximize anatomical visibility, minimize intraoperative risks, and ensure standardization across cases and surgeons. Our technique incorporates early vascular control, strategic ureteral visualization, and layered vaginal closure, each supported by prior high-quality studies. Compared to other series, our clinical outcomes—especially operative time, blood loss, and complication rates—are comparable to or better than the published norms, reinforcing the practical effectiveness of our approach. We believe that the implementation of such a stepwise system may accelerate training in laparoscopic gynecology by providing a reproducible and teachable framework, improve the consistency and transparency of surgical audits across institutions, and ultimately reduce the variability in complication rates associated with TLH. Future multicenter prospective studies and randomized trials may further refine or validate these standardized steps and assess long-term outcomes such as pelvic floor support and sexual function. We believe that a structured, anatomically grounded protocol can support surgical education and reduce the variability associated with the learning curve of laparoscopic hysterectomy.

We believe that a structured, anatomically grounded protocol can support both surgical education and clinical practice by reducing variability and improving reproducibility. While primarily designed as a training tool, its utility extends to most routine TLH cases, with flexibility for adaptation in more complex situations.

## Figures and Tables

**Table 1:** Stepwise anatomy-based protocol for total laparoscopic hysterectomy.

Step No.	Step Name	Brief Description	Key Anatomical/Technical Points
1	Right pelvic sidewall dissection	Round ligament transection, retroperitoneal entry, uterine artery ligation, fenestration of the broad ligament above the ureter, and transection of the mesosalpinx and ovarian ligament (or infundibulopelvic ligament in case of adnexectomy)	Uterine artery ligated at origin from internal iliac artery; ureter identified and protected; broad ligament fenestration improves exposure; IP ligament handled cautiously to avoid ureteral injury
2	Left pelvic sidewall dissection	Same as step 1, mirrored	Symmetric approach ensures consistent ureteral visualization
3	Bladder mobilization	Vesicouterine space developed via anterior peritoneal fold dissection	Blunt dissection; paravesical space used in scarred patients
4	Posterior peritoneum & USL dissection	Uterosacral ligament transection with optical rotation	Enhances exposure to deep pelvis; switch of dominant instrument hand improves ergonomics
5	Cardinal ligament dissection	Transection of cardinal ligament and distal uterine artery	Performed under direct traction of manipulator for safety
6	Colpotomy	Circumferential incision around cervix	Posterior to anterior; monopolar cutting for minimal thermal damage
7	Uterus extraction	Removal via vagina or morcellation as needed	Depends on size; vaginal preferred if feasible
8	Vaginal cuff closure	Laparoscopic figure-of-eight intracorporeal suturing	Includes mucosa, fascia, and uterosacral ligaments for support

**Table 2:** Patient demographics and surgical history.

Patients (*n* = 109)	Mean ± SD or Count
Age (years)	51.1 ± 10.3
BMI	26.8 ± 5.3
BMI >30 (*n*,%)	19 (17.4)
Parity: 1–4 (*n*,%)	99 (90.8)
Postmenopausal status (*n*,%)	36 (33)
History of vaginal delivery (*n*,%)	88 (80.7)
Previous laparoscopic surgery (*n*,%)	19 (17.4)
Previous open surgery (*n*,%)	38 (34.9)

BMI: body mass index; SD: standard deviation.

**Table 3:** Summary of surgical and perioperative parameters.

Patients (*n* = 109)	Value (±SD)
Indication: Fibroids *n*, (%)	35 (32.1)
Indication: Abnormal uterine bleeding *n*, (%)	43 (39.5)
Indication: Other *n*, (%)	31 (28.4)
Uterine weight (g)	211.9 (±95.3)
Total operative time (min)	67.2 (±18.3)
Preoperative hemoglobin (g/dL)	12.5 (±1.5)
Hemoglobin drop on POD-1 (g/dL)	1.2 (±0.9)
Hospital stay (days)	2.49 (±1.14)
Prolonged hospital stay >4 days, *n* (%)	10 (9.2)

SD: standard deviation, POD-1: first postoperative day.

**Table 4:** Intraoperative and postoperative complications.

Complication Type	*n* (%)
Bladder injury	2 (1.8)
Small bowel injury	2 (1.8)
Vaginal cuff dehiscence	2 (1.8)
Vaginal evisceration	0 (0)
Vaginal cuff hematoma/abscess	1 (0.9)
Postoperative vaginal bleeding	4 (3.7)
Postoperative infection (non-surgical)	0 (0)
Reoperation	1 (0.9)
Laparoconversion	0 (0)
Blood transfusion	2 (1.8)
Paralytic ileus	2 (1.8)

## Data Availability

The data presented in this study are available by contacting the corresponding author.

## Supplementary Materials

The following Supporting Information can be downloaded at: https://drive.google.com/file/d/1XB0v5uOAtIybUPzcmoqb6pZzpufvPou9/view?usp=sharing, Video S1: Standardized surgical steps of TLH.
